# “The many faces of sorrow”: An empirical exploration of the psychological plurality of sadness

**DOI:** 10.1007/s12144-023-04518-z

**Published:** 2023-04-13

**Authors:** Myron Tsikandilakis, Persefoni Bali, Zhaoliang Yu, Alexandros-Konstantinos Karlis, Eddie Mun Wai Tong, Alison Milbank, Pierre-Alexis Mevel, Jan Derrfuss, Christopher Madan

**Affiliations:** 1 Medical School, Faculty of Medicine and Health Sciences, University of Nottingham, Nottingham, UK; 2 School of Psychology, University of Nottingham, Nottingham, UK; 3 Department of Psychology, Wuhan University, Wuhan, China; 4 Department of Physics, National and Kapodistrian University of Athens, Athens, Greece; 5 Department of Psychology, National University of Singapore, Singapore, Singapore; 6 Department of Theology and Religious Studies, University of Nottingham, Nottingham, UK; 7 Department of Modern Languages and Cultures, University of Nottingham, Nottingham, UK

**Keywords:** Sadness, Emotional states, Faces, Scenes, physiology

## Abstract

Sadness has typically been associated with failure, defeat and loss, but it has also been suggested that sadness facilitates positive and restructuring emotional changes. This suggests that sadness is a multi-faceted emotion. This supports the idea that there might in fact be different facets of sadness that can be distinguished psychologically and physiologically. In the current set of studies, we explored this hypothesis. In a first stage, participants were asked to select sad emotional faces and scene stimuli either characterized or not by a key suggested sadness-related characteristic: loneliness or melancholy or misery or bereavement or despair. In a second stage, another set of participants was presented with the selected emotional faces and scene stimuli. They were assessed for differences in emotional, physiological and facial-expressive responses. The results showed that sad faces involving melancholy, misery, bereavement and despair were experienced as conferring dissociable physiological characteristics. Critical findings, in a final exploratory design, in a third stage, showed that a new set of participants could match emotional scenes to emotional faces with the same sadness-related characteristic with close to perfect precision performance. These findings suggest that melancholy, misery, bereavement and despair can be distinguishable emotional states associated with sadness.

## Introduction

From Burton’s eloquent *Anatomy of Melancholy* (1845/1989) to Beethoven’s furious sorrow in *The Tempest* (1802/2007) or Schopenhauer’s existential nihilism in *The World as Will and Representation* (1819/2012), sadness has engaged the attention of some of the greatest minds in human history (Allister, 2001). Aristotle, in his *Metaphysics* (350 B.C.E./ 1999), relates a relevant incidence (see Witt, 2018; Judson, 2019). He recollects being a young man at the riverbanks of ancient Athens. He is examining the physical properties of water and earth for several hours when a feeling of harrowing angst starts to overcome him. He speculates that this experience must not be a constituent of the elements he is examining. It must be a personal biological reaction. He speculates his blood must have a temporary excess of black bile (black: *μέλας*, bile: *χολή*; melancholy: *μελαγχολία*), that can be the outcome of various and diverse elicitors, such as prolonged loneliness, the loss of a family member and unreciprocated longing for a lover (Jouanna, 2012). The feeling overwhelms him, and he makes his way to the *agora* to alleviate his melancholy (Konstan, 2006). In his story, Aristotle provides us with observations which Sigmund Freud (1917/2005), William James (1894) and Schachter and Singer (1962) would emphasize more than 2,000 years later: Sadness is an emotional experience with biological correlates, it does not necessarily have a singular elicitor and signals the need for cognitive and behaviour-changing coping processes (Power, 2010).

In contemporary psychological science (Lomas, 2018), sadness is considered one of the six basic emotions (i.e., anger, disgust, fear, happiness, sadness surprise; Ekman & Friesen, 1972), and it is considered a universal emotion (Ekman, 1999; Ekman, 2004; Tracy & Randles, 2011; see also Barrett et al., 2019). This can be interpreted to mean that sadness has certain characteristics. Sadness involves specific central and peripheral nervous system physiological correlates. It involves specific elicitors which contribute to the experience of sadness. It involves specific facial-expressive movements and should be encountered cross-culturally (but see also Jackson et al., 2019) because it serves evolutionary important communication purposes (Cabanac, 2002).

These properties are suggested to occur due to the function of sadness as an emotional state. The basic proposed function of sadness is to provide adaptive coping mechanisms that contribute to personal reflection after the loss of an important subject or object (Lazarus, 1991; Keedwell, 2008; Power, 2010; for a thorough overview, see Barrett et al., 2019). This is suggested to involve a decrease in peripheral physiological arousal (Shirai & Suzuki, 2017). It is suggested to include the activation of neural structures involved in processing emotions and carrying out executive functions (Phan et al., 2002; Murphy et al., 2003).

These responses are suggested to enable an individual to “come to terms” with the experienced loss, plan and – if necessary – revise their cognitive and behavioural attitudes and strategies (Power, 2010). The facial display of sadness is suggested to involve cross-culturally homogeneous (Ekman & Friesen, 1972) lowering brow (Action Units 1 & 5) and mouth movements (Action Units 15 & 17; Ekman, 2004). These are suggested to communicate muscular depression of the facial structure. Their aim is to express personal discontent, attract interpersonal sympathy and signal the need for support, such as emotional care and the provision of practical assistance (Reed & DeScioli, 2017).

One issue in psychological research in this area is that perspectives are conflicting as regards the proposed physiological and social-emotional functions associated with sadness (Arias et al., 2020). Many contemporary psychologists consider that the characteristics of sadness are the most unclear amongst those of the six basic emotions (Ekman, 1992a, b, 1999; see also Collet et al., 1997; Palomba et al., 2000; Rainville et al., 2006; Vytal & Hamann, 2010; Saarimäki et al., 2018).

For example, sadness is suggested to induce a decrease in peripheral nervous system arousal to enable introspection and self-reflection (Welling, 2003). This is suggested to enable an increase in analytic thinking abilities (Overskeid, 2000). Sadness is also suggested to elicit a reduction of false-memory biases during autobiographical recall (Storbeck & Clore, 2005). Contrary to these, multiple studies have shown that the presentation of sad emotional elicitors leads to an increase in skin conductance responses (SCR) and heart-rate responses (HR) (Banks et al., 2012; Robinson & Demaree, 2009). Several studies have shown that sadness induced by negative performance, such as failure to achieve in an engagement task (Rottenberg & Gross, 2003; Rottenberg et al., 2003), and presented sad faces that involved crying, such as tearful faces, led to increased SCR and HR (Shirai & Suzuki, 2017).

Listening to sad music has been shown to elicit positively valanced psychophysiological correlates, such as increase in skin conductance that participants characterized as euphoric (Sachs et al., 2015). In our own studies, on the unconscious processing of sadness, hits for brief masked sad faces (i.e., post-trial self-reports that a masked sad face was presented when a masked sad face was presented) repeatedly and consistently showed a significant increase in physiology, such as SCR and HR (Tsikandilakis & Chapman, 2018; Tsikandilakis et al., 2018; Tsikandilakis et al., 2019; Tsikandilakis et al., 2019a; Tsikandilakis et al., 2019b; Tsikandilakis et al., 2020a, 2020b; 2020c; Tsikandilakis et al., 2021a; 2021b; 2021c; Tsikandilakis et al., 2022a; 2022b; 2022c; 2022d).

Sadness has also been linked with prosocial and socially-adaptive behaviours (Forgas, 2017). Interestingly, it has also been linked with a desire for social withdrawal and social self-exclusion (Duijndam et al., 2020). It has been linked with higher autobiographical recall but a decrease in working and short-term memory systems (Storbeck & Maswood, 2016). Sadness has also been associated with a significant increase of the anchoring bias, meaning in this context that individuals who experience sadness often default to the original explanation of a phenomenon even if the explanation was initially characterized as an insufficient explanation for that phenomenon (Bodenhausen et al., 2000). These indicate that if sadness is indeed “the architect of cognitive change” (Karnaze & Levine, 2018), it functions in a multiplicity of ways (Arias et al., 2020).

Based on these findings, our exploratory hypothesis was that there could be several emotional states – with discernible physiological and eliciting characteristics – which are associated with sadness. We expected these emotional states to provide evidence for variations in intensity and valence. We took particular care to explore whether different emotional states with similar ratings for intensity and valence involved different physiological responses and eliciting circumstances, such as different types of eliciting events, skin conductance and heart-rate responses, to test our findings for showing non-discernible linear escalations of prototypical sadness. In the same manner, we also explored whether lower and higher in intensity and valence emotional states involved dissociable types of elicitors.

We chose to explore the physiological and emotional characteristics of several emotional concepts: melancholy, loneliness, misery, bereavement and despair. We chose these emotional concepts because previous reviews have repeatedly, consistently and with emphasis prioritized them as potential rally points for further exploring sadness. These have been suggested to be associated with sadness and to involve distinguishable eliciting and physiological correlates (Allister, 2001; Arias et al., 2020; Barrett et al., 2019; Bodenhausen et al., 2000; Brown et al., 1995; Diener et al., 2012; Forgas, 2017; Hariri et al., 2002; Karnaze & Levine, 2018; Keedwell, 2008; Lomas, 2018; Maj, 2008; Power, 2010; Reed & DeScioli, 2017; Robinson & Demaree, 2009; Sachs et al., 2015; Shirai & Suzuki, 2017; Welling, 2003).

In the first stage of our research, we used participant assessment to select emotional faces and International Affective Picture System (IAPS; Lang et al., 1997) images related to these states. This stage included two phases. In one phase, faces were selected. In phase two scenes from the IAPS were selected. The stimuli were selected for use in subsequent experimental stages. In a second stage, we presented participants with the selected emotional faces and IAPS images and measured SCR, HR, facial-emotional characteristics and responses, and self-reports for the experience of these emotional states. The exploratory aim of this stage was to investigate whether sadness involving different characteristics could confer results for dissociable behavioural and psychophysiological responses. Finally, in a third stage, we explored the validity of our results using a novel design. We explored whether participants could accurately match IAPS images as possible elicitors of emotional faces which were labelled as expressing the same emotional state associated with sadness. Our overall objective was to explore whether we could provide empirical evidence for the existence of psychological plurality in the expression of sadness.

## Stage one: Face and scene selection

### Phase one: Selection and assessment of emotional faces

#### Aims

The aim of this phase of stage one was to select faces expressing melancholy, loneliness, misery, bereavement and despair from an existing dataset. We assessed via participant engagement question tasks whether these faces displayed differences for the expression of emotion. Our exploratory hypothesis for this phase was that empirical evidence could be revealed for differences between the presented emotional images.

#### Participants

A power calculation based on medium effect sizes (η^2^_p_ = 0.06; f = 0.25) and within-subject experimental trial repetitions was performed (see, particularly, Faul et al., 2009). The result revealed that fifty-two participants would be required for P _(1-β)_ ≥ 0.9; (*p* ≤ 0.05; P (H_0_) ≥ 0.9; B < 0.33; η^2^_p_ [0, < 0.001]) (Kelter, 2021). Fifty-five volunteers participated in this phase. The exclusion criteria were that participants must not have been previously exposed to the emotional database for this phase (Gur et al., 2002). Participants must not have a current or previous DSM-5 Axis I or II diagnosis (American Psychiatric Association, 2013) and be experiencing or have experienced a serious personal loss, or serious or debilitating non-clinically assessed depressive mood or moods in the last six months via self-report (see, particularly, Maj, 2008). To confirm the self-reports using a standardised assessment for depression and psychiatric diagnoses, the participants were screened with the Somatic and Psychological Health Report Questionnaire (SPHRQ; Berryman et al., 2012; Hickie et al., 2001). To confirm the self-reports using a standardised assessment for the experience of recent depression-related traumatic events, participants were assessed with the Stressful Life Events Screening Questionnaire (SLESQ; Allen et al., 2015; Goodman et al., 1998; Gray et al., 2004). To confirm that participants could appraise the emotional context of the presented emotional faces, they were assessed with an on-line alexithymia questionnaire (Alexithymia, 2020; see also, Ridout et al., 2021). One participant was excluded from the analysis due to having above baseline scores for Alexithymia sub-traits indicating possible alexithymia traits (M _emotional-recognition_ ≥ 27). Two participants were excluded from the analysis due to the loss of close family member in the past six months. The final population sample consisted of fifty-two participants (twenty-six female) with mean age 27.81 (SD = 5.23). The experiment was approved by the Ethics Committee of the School of Psychology of the University of Nottingham.

#### Facial stimuli

The sad facial stimuli used were taken from the dataset created by Gur and colleagues (2002). In this dataset, actors were asked to remember a personal emotionally significant event associated with each basic emotion and express facial responses freely and subjectively while multiple photographs for each actor and emotion (≥5) were taken (Gur et al., 2002; 139–141; see supplementary material 1.1). Faces in the dataset were adjusted for interpupillary distance, transformed to grey scale and downsized to a standard 1024 × 768 pixels resolution. Their luminescence was averaged in SHINE, MATLAB Toolbox and finally the faces were spatially aligned and framed into pure white within a cropped circle (Height: 6 cm, Width: 4 cm).

#### Participant assessment

**Fig. 1:**
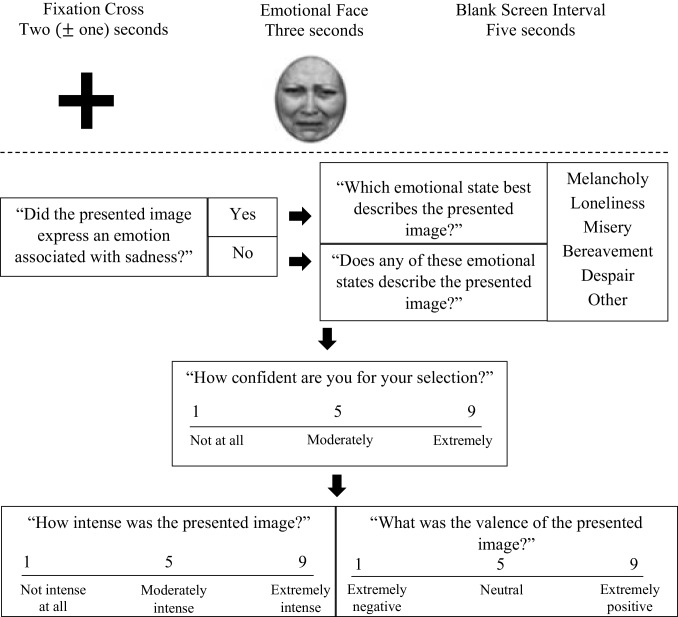
Experimental sequence for stage one, phase one. Participants were presented with a fixation cross for two (± one-second) and subsequently an emotional face for three seconds. The duration of the emotional face was implemented to last for three seconds to enable participants to thoroughly evaluate the presented stimulus. After the presentation, a blank screen was shown and consequently participants were assigned a series of engagement question tasks

The stimuli for this phase were presented on an HD high frequency LED monitor (144 Hz) and the presentation was created in the Builder and Coder components of PsychoPy v.1.90.02 (Peirce, 2007). Three-hundred freely-expressed faces labelled as sad with intensity ratings ranging from one (mild) to three (moderate) from one-hundred actors (fifty female) were presented in two same-day sessions divided by a five-minute rest break. The participants were unaware of the pre-labelled dataset intensity, and they were not informed concerning the dataset ratings at any part of the experiment. They were asked in the beginning of each session by an on-screen message to reply to the experimental questions freely and according to their own subjective perception. The experiment started with a training stage during which participants familiarised themselves with the keyboard and mouse response components and the terminology of the experiment. The main experiment started with a fixation cross for two (± one-second). After the fixation cross, a single face was presented at fixation for three seconds. A blank screen interval was then presented for five seconds (see Stage Two: *Physiological Assessment*). After the interval participants were asked “Did the presented image express an emotion associated with sadness?”. The participants were asked to use the keyboard to choose “yes” (a) or “no” (s); the key assignment and the order of the placement of the two choices was randomised in each trial (see supplementary material 2.1, 2.2 & 2.3). After this question we used conditional branching. If the participant replied “yes” they were asked “Which emotional state best describes the presented image?”. They were asked to choose a single item from an on-screen list using the keyboard. The options included “melancholy” (a), “loneliness” (s), “misery” (d), “bereavement” (z), “despair” (x) and “other” (c); the key assignment and the order of the list was randomised in each trial. If the participants replied “no” they were asked “Does any of these emotional states describe the presented image?”. The available responses and randomization parameters for this condition were identical with the first branching condition. This question was included to avoid shortcut response biases (see Wetzel et al., 2016; see also supplementary material 3). After the conditional-branching task, participants were asked to use the mouse to rate from one (not at all) to nine (extremely) the confidence for their selection and press OK to confirm their choice. After the confidence responses, participants were asked to answer two additional questions with order randomised. They were asked to use the mouse to rate from one (not intense at all) to nine (extremely high) the intensity of the emotional expression and press OK to confirm their choice. They were asked to use the mouse to rate from one (extremely negative) to nine (extremely positive) the valence of the emotional expression and press OK to confirm their choice. A two-second blank screen was presented before the next trial (see Fig. 1).

#### Stimulus selection

**Fig. 2:**
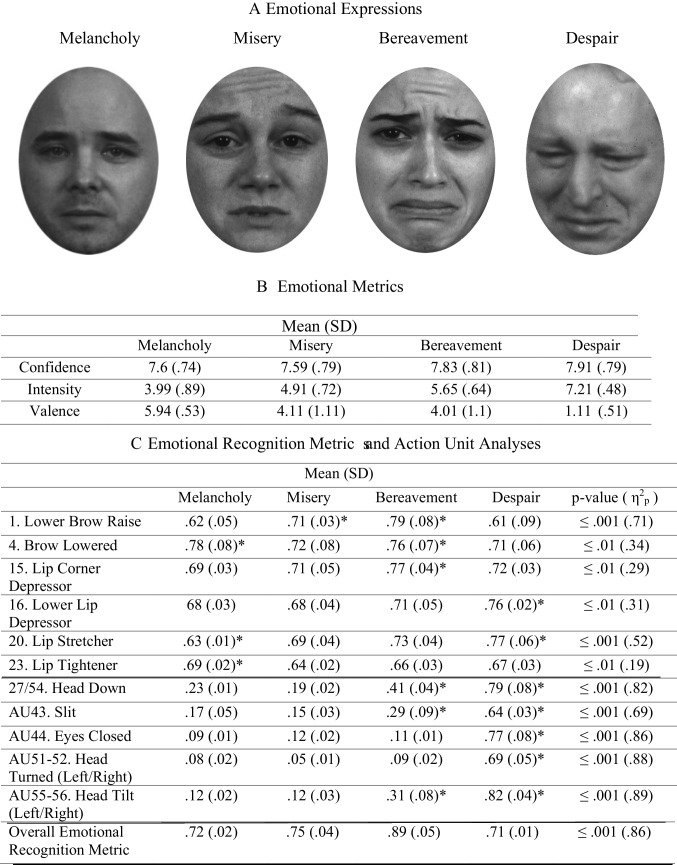
Emotional states, stage one, phase one. In **A**. example faces for each emotional state. In **B**. emotional metrics for each emotional state including mean and standard deviation. In **C**. the emotional recognition metric for action units 1, 4, 15, 16, 20 and 23 that have previously been associated with the expression of sadness. In the right of the screen in C. ANOVA p-values and effect size η^2^ for each reported action unit category/row. Asterisks indicate Bonferroni corrected significance for post hoc comparisons at *p* ≤ .01. Also, in C. the explorative assessment of units 27/54, AU43, AU44, AU51 and 52 and AU55 and 56 that are not commonly associated with sadness (Arias et al, 2020) but were associated with despair after post-hoc analyses of the facial-expressive outcome characteristics (for format, see American Psychiatric Association, 2019). These findings suggested that despair in particular could relate to specific head movements that are not encountered in other emotional states associated with sadness

The faces which participants chose as associated with sadness and with 100% agreement for expressing melancholy (n = 29), loneliness (n = 7), misery (n = 31), bereavement (n = 28) and despair (n = 33) were selected. We aimed to select twenty faces per emotional state and, therefore, loneliness was excluded from further analyses. For the four categories, faces were further selected to control for actor repetition and to include an equal number of males and female actors. Finally, from the selected pool (n = 91) the faces with the higher confidence for selection were chosen for the four categories. The final set included eighty faces from forty actors (twenty female; see Fig. 2) showing melancholy, misery, bereavement and despair (n _category_ = 20). All included faces were rated as moderate in intensity within the Gur and colleagues (2002) database (see also supplementary material 1 & 2.1–2.3). No actor identity was repeated. When a face was selected for more than one emotional state, the face was assigned to the emotional state that involved the highest confidence for emotional selection for that face.

#### Facial recognition software

As a validation measure, we used computer-based analysis of the resulting pool of images using Noldus FaceReader 7.1. The analysis employed the Viola-Jones cascaded algorithm and an active appearance model (AAM) to eliminate static identification variability. The analysis included the in-built emotional categorization labels included in Noldus (anger, fear, surprise, happiness, sadness and neutral) and a percentage-based assessment metric measuring the extent to which facial action units were activated resulting in an overall percentage-based emotional-recognition metric for the identification of emotion which indicated how pronounced an emotion was in the assessed face (Lewinski et al., 2014). The range of the assessment metric was 0 to 1 with 0.6 signifying the baseline for a categorical recognition of an emotion or action unit activation and scores ≥ 0.6 signifying progressively more prototypical expressions of emotional expressions (see Skiendziel et al., 2019; see Fig. 2).

#### Results and discussion

**Fig. 3:**
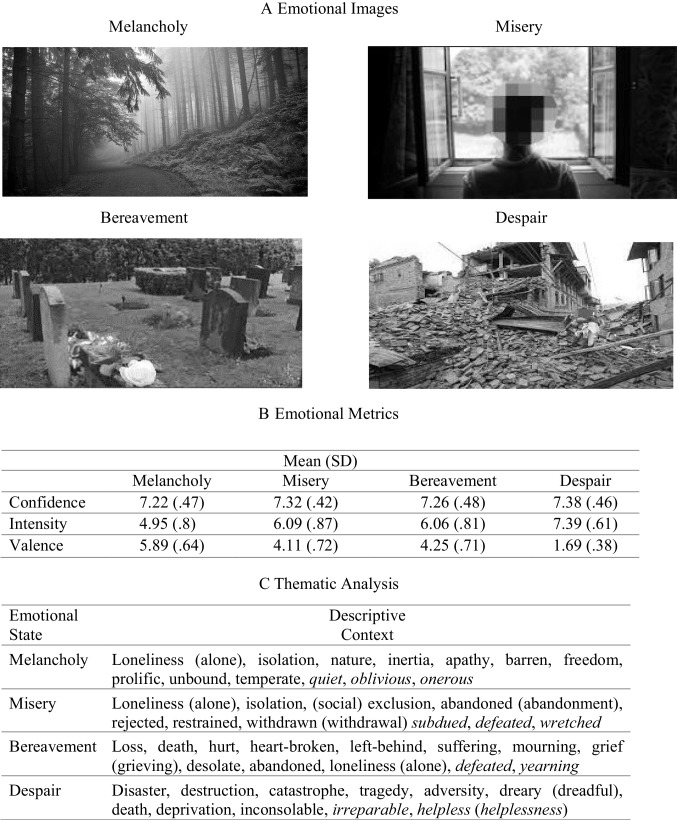
Emotional images, stage one, phase two. In **A**. example images for each emotional state. The images are not IAPS stimuli due to copyright restrictions. In **B**. emotional metrics for each emotional image type including mean and standard deviation. In **C**. qualitative thematic analysis of the final pool of images (Colden et al., 2008; Guest et al., 2011; Constantinescu et al., 2017) Contextual descriptions used multiple times (≥5) for more than one image (≥2) for one emotional state are presented in *italics*. Contextual descriptions that described an already existing emotional label were triaged

The analysis was performed using significance testing and Bayesian statistics. For every non-significant finding, a Bayes factor was calculated using the Dienes Calculator with credible intervals set at two standards errors of the mean or combined variable means of the outcome variables and evidence for the null set at B < 0.33, insensitivity for both hypotheses set at 0.33 ≤ B ≤ 3 and evidence for the alternate hypothesis set at B > 3 (see Dienes, 2016). To explore whether there were differences in confidence for selecting an emotional state, a repeated measures ANOVA was run with independent variable Type of Emotion (Melancholy, Misery, Bereavement & Despair) and dependent variable confidence ratings. The analyses revealed a trend for differences between emotional states for confidence (F (3, 153) = 2.31; *p* = 0.08; η^2^_p_ = 0.04; SE = 0.06; B = 2.89). Significant differences were revealed between emotional states for intensity (F (2.51, 127.89) = 114.69; *p* < 0.001; η^2^_p_ = 0.79; Greenhouse-Geisser corrected; SE = 0.05; B =  + ∞). Further Bonferroni corrected comparisons revealed that despair was higher for intensity compared to melancholy (*p* < 0.001; d = 4.51), misery (*p* < 0.001; d = 3.72) and bereavement (*p* < 0.001; d = 2.78). Misery (*p* < 0.001; d = 1.13) and bereavement (*p* < 0.001; d = 2.13) were higher than melancholy for intensity ratings. Bereavement was higher for intensity compared to misery (*p* < 0.001; d = 1.77). Similar findings were revealed for ratings for valence (F (2.32, 118.28) = 198.03; *p* < 0.001; η^2^_p_ = 0.79; Greenhouse-Geisser corrected; SE = 0.06; B =  + ∞). Bonferroni corrected comparisons revealed that despair was rated for having more negative valence compared to melancholy (*p* < 0.001; d = 9.27), misery (*p* < 0.001; d = 2.32) and bereavement (*p* < 0.001; d = 2.24). Misery (*p* < 0.001; d = 3.28) and bereavement (*p* < 0.001; d = 3.36) were more negative for valence compared to melancholy. Bereavement and misery were not significantly different (*p* = 0.98; d = 0.06) and provided Bayesian evidence for proximate ratings for valence (SE = 0.15; B = 0.09 l see Fig. 3). No gender or session sequence effects were reported for comparisons for confidence, intensity and valence (see supplementary material 4). All faces were recognized as expressing sadness using facial-emotional recognition software analyses. Further quantitative analysis for the Noldus emotional recognition metric for the identification of emotion revealed differences between melancholy, misery, bereavement and despair (F (1.38, 29.07) = 124.18; *p* < 0.001; η^2^_p_ = 0.86; SE = 0.01; B =  + ∞). Further Bonferroni corrected comparisons revealed that bereavement was higher for emotional recognition than melancholy (*p* < 0.001; d = 4.79), misery (*p* < 0.001; d = 3.09) and despair (*p* < 0.001; d = 4.88). These findings suggested that the assessed emotional states illustrated discernible differences for self-report ratings and facial-emotional expressive characteristics that were not linear escalations of prototypical sadness (see Fig. 2).

### Phase two: Selection and assessment of IAPS images

#### Aims

The aim of this phase of stage one was to select from the International Affective Picture System (IAPS) images which are associated with sadness such as melancholy, misery, bereavement and despair. We assessed via participant engagement question tasks whether these images displayed differences for emotional characteristics. Our exploratory hypothesis for this phase was that there could be reported differences between images related to each emotional state.

#### Participants

Fifty-three volunteers participated in this phase. The exclusion criteria were identical with phase one. One participant was excluded from the analysis due to the loss of a close family member in the past six months. The final population sample consisted of fifty-two participants (twenty-seven female; P _(1-β)_ ≥ 0.9; see Stage One: *Participants*). The mean age of the participants was 29.06 (SD = 3.17). No participant from phase one was included in phase two. The experiment was approved by the Ethics Committee of the School of Psychology of the University of Nottingham.

#### Image stimuli

The image stimuli used were taken from the IAPS database (Lang et al., 1997). Three-hundred IAPS images that received ethical approval for presentation in this phase and were associated with sadness, such as high and low intensity and negative valence, and explicit categorizations for evoking sad emotions, in previous empirical research were included (see Mikels et al., 2005; Libkuman et al., 2007; Lang & Bradley, 2007). These images were not allowed to show violence, legal violations, human and animal harm, and abuse, sexual and erotic scenes, and they did not portray explicit or implicit threat (IAPS image codes can be found in supplementary material 6; see also Fig. 3C). The images were transformed to grey scale, downsized to a standard 1024 × 768 pixels resolution and their luminescence was averaged in SHINE, MATLAB Toolbox. All images were transformed to visual vectors and facial expressions, gender characteristics and emotional bodily postures were pixelated to avoid parallel processing (see Colden et al., 2008). The processed vectors were spatially re-integrated in the original picture and further adjusted for luminescence using CorelDRAW Graphics Suite 2021. The vectorized images were, finally, framed in pure white within a set-dimensions cropped parallelogram (Height: 6 cm, Width: 8 cm; see Lang & Bradley, 2007).

#### Participant assessment

The stimuli for this phase were presented using the same basic instructions, equipment and apparatus and coded in the same platform as phase one. Three-hundred images were presented in two same-day sessions divided by a five-minutes rest break. The experiment started with a training stage during which participants familiarised themselves with the keyboard and mouse response components and the terminology of the experiment. The experimental sequence was identical with phase one (see Fig. 1).

#### Stimulus selection

The images which participants chose as associated with sadness and with 100% agreement for expressing melancholy (n = 31), misery (n = 29), bereavement (n = 32) and despair (n = 26) were selected. We aimed to select twenty images per emotional state. For the four categories, images were further selected based on confidence for selection. The final set included eighty images showing melancholy, misery, bereavement and despair (n _category_ = 20; for IAPS image codes, see supplementary material 6). No stimulus was selected more than once. When a stimulus was selected for more than one emotional state, the stimulus was assigned to the emotional state that included the highest confidence for emotional selection for that stimulus.

#### Results and discussion

To explore for differences in confidence, a repeated measures ANOVA was run with independent variable Type of Emotion (Melancholy, Misery, Bereavement & Despair) and dependent variable confidence ratings. The analyses did not reveal differences between image types (F (3, 153) = 1.42; *p* = 0.24; η^2^_p_ = 0.02; SE = 0.04; B = 1.89). Significant differences were revealed between emotional images for intensity (F (3, 151) = 95.35; *p* < 0.001; η^2^_p_ = 0.65; SE = 0.06; B =  + ∞). Further Bonferroni corrected comparisons revealed that despair was higher for intensity compared to melancholy (*p* < 0.001; d = 3.44), misery (*p* < 0.001; d = 1.72) and bereavement (*p* < 0.001; d = 1.86). Misery (*p* < 0.001; d = 1.35) and bereavement (*p* < 0.001; d = 1.38) were higher than melancholy for intensity ratings. Bereavement and misery were not different for intensity (*p* = 0.99; d = 0.02) and provided Bayesian evidence for proximate ratings (SE = 0.16; B = 0.07). Similar findings were revealed for ratings for valence (F (2.47, 125.78) = 353.89; *p* < 0.001; η^2^_p_ = 0.87; Greenhouse-Geisser corrected; SE = 0.04; B =  + ∞). Further Bonferroni corrected comparisons revealed that despair was rated for having more negative valence compared to melancholy (*p* < 0.001; d = 8.02), misery (*p* < 0.001; d = 4.28) and bereavement (*p* < 0.001; d = 4.53). Misery (*p* < 0.001; d = 2.63) and bereavement (*p* < 0.001; d = 2.43) were more negative for valence compared to melancholy. Bereavement and misery were not significantly different (*p* = 0.98; d = 0.19) and provided a trend for Bayesian evidence for proximate ratings for valence (SE = 0.15; B = 0.47). No gender effects or session sequence effects were reported for comparisons for confidence, intensity and valence (see supplementary material 5). These findings suggested that the assessed emotional images related to melancholy, misery, bereavement and despair showed discernible differences for self-report ratings for emotional characteristics and – once more – that the selected images were not linear escalations of prototypical sadness (see Fig. 3; see also details for the Thematic Analysis for this phase in supplementary material 6).

### Stage two: Physiological assessment

#### Aims

The aim of stage two was to assess participants using ratings, skin conductance (SCR), heart rate (HR) and facial-emotional recognition metrics for responses to the selected emotional faces, and IAPS images that were associated with melancholy, misery, bereavement and despair. We wanted to explore whether different emotional states were associated with different physiological responses.

#### Participants

A power calculation based on medium effect sizes (η^2^_p_ = 0.06; f = 0.25) and within-subject experimental trial repetitions was performed. The result revealed that sixty-seven participants would be required for P _(1-β)_ ≥ 0.9; (*p* ≤ 0.05; P (H_0_) ≥ 0.9; B < 0.33; η^2^_p_ [0, < 0.001]). The exclusion criteria for this stage were identical with phases one and two in stage one. Seventy-one volunteers participated in this stage. Data from two participants were excluded from the analyses due to the loss of a close family member in the last six months. Data from one participant was excluded due to possible alexithymia traits. Data from one participant were excluded due to SPHERE-12 scores (> 3) that indicated a possible psychiatric diagnosis. The final sample consisted of sixty-seven participants (thirty female) with average age 27.44 (SD = 3.21). No participant from stage one was included in stage two. The experiment was approved by the Ethics Committee of the School of Psychology of the University of Nottingham.

#### Physiological assessment

Skin conductance and heart rate were used to assess physiological responses. Skin-conductance responses were measured from the left hand (index/first and middle/second fingers) of each participant using disposable Ag/AgCl gelled electrodes. The signals were received by a BIOPAC System, EDA100C in units of microsiemens (μS) and recorded in AcqKnowledge (Braithwaite et al., 2013). Heart rate was measured via a single finger sensor from the left hand (ring/third finger). The signal was measured by a BIOPAC System, PPG100C using infra-red photoplethysmogramy of blood flow fluctuations and converted and recorded in beats per minute (bpm) in AcqKnowledge. The occurrence of a phasic skin-conductance response was defined as an unambiguous increase (0.01μS) with respect to each pre-target skin-conductance baseline score occurring up to three seconds post stimuli offset (van der Ploeg et al., 2017). The occurrence of a heart-rate response was defined as an event-related heart rate peak in beats per minute with respect to each pre-target heart-rate baseline score occurring up to five seconds post stimuli offset (Cacioppo et al., 2007; pp. 182–189).

#### Facial recognition software

Computer-based analysis of emotional responses was conducted using Noldus FaceReader 7.1. The analysis was conducted using an HD camera mounted on the bottom of the presenting screen and centred on the participant’s face. The analysis was run using the maximum video capture frames per second allowed by the face-reader equipment (thirty fps). Each participant was evaluated in respect to the expressed emotion after controlling for the influence of action units which were present in their own neutral expressions using the participant calibration module (Noldus, 2021). The analysis included the in-built emotional categorization labels included in Noldus (anger, fear, surprise, happiness, sadness and neutral). Facial-emotional recognition of an emotion was defined as a categorical classification of an emotional response up to five seconds post-stimuli offset. Participants were aware that their facial expressions were being recorded.

#### Participant assessment

The stimuli for this phase were presented using the same equipment and apparatus and coded in the same platform as stage one. Eighty emotional faces and eighty IAPS images (n _emotional state_ = 20) were presented in two same-day sessions divided by a five-minute rest break with order randomised. The experiment started with a training stage during which participants familiarised themselves with the response components and the terminology of the experiment. The main experiment started with a fixation cross for two (± one) seconds. After the fixation cross a single emotional face or scene showing melancholy, misery, bereavement or despair was presented at fixation for three seconds. A blank screen interval was then presented for five seconds. Physiological and facial-emotional responses were assessed during this interval (Cacioppo et al., 2007; pp. 161–163 & 182–189). After the interval participants were assigned an engagement task to ensure they were paying attention to the presentation. They were asked to use the mouse to answer whether a scene or a face was presented. The data for the attention-engagement task were not analysed further. After the engagement task a blank screen interval was presented for seven seconds to allow physiological responses to return to baseline before the next trial.

#### Results and discussion: Emotional faces

To explore whether there were differences in physiological changes, a repeated measures ANOVA was run with independent variable Type of Emotion (Melancholy, Misery, Bereavement & Despair) and dependent variable SCR. The analyses revealed there were differences in SCR changes between the emotional states (F (2.56, 169.09) = 267.42; *p* < 0.001; η^2^_p_ = 0.8; Greenhouse-Geisser corrected; SE = 0.01; B =  + ∞). Further Bonferroni corrected comparisons revealed that despair was higher for SCR compared to melancholy (*p* < 0.001; d = 4.39), misery (*p* < 0.001; d = 2.09) and bereavement (*p* < 0.001; d = 2.22). Misery (*p* < 0.001; d = 2.89) and bereavement (*p* < 0.001; d = 2.72) were also higher than melancholy for skin conductance responses. Misery and bereavement did not provide evidence for being significantly different (*p* = 0.98; d = 0.18) and provided a trend for Bayesian evidence for proximate SCR changes (SE = 0.01; B = 0.78). Similar results were revealed for heart-rate responses and facial-emotional recognition responses. For heart-rate responses there were significant differences between the emotional states (F (2.45, 161.67) = 270.64; *p* < 0.001; η^2^_p_ = 0.81; Greenhouse-Geisser corrected; SE = 0.05; B =  + ∞). Bonferroni corrected comparisons revealed that despair was higher for HR compared to melancholy (*p* < 0.001; d = 4.11), misery (*p* < 0.001; d = 2.58) and bereavement (*p* < 0.001; d = 2.56). Misery (*p* < 0.001; d = 2.14) and bereavement (*p* < 0.001; d = 2.16) were higher than melancholy. Misery and bereavement did not provide evidence for being significantly different and provided Bayesian evidence for proximate HR changes (*p* = 0.99; d = 0.02; SE = 0.11; B = 0.78). For facial-emotional recognition, participants exhibited expressions of sadness involving significant differences for the emotional metric associated with emotional recognition (F (2.46, 162.49) = 55.95; *p* < 0.001; η^2^_p_ = 0.46; Greenhouse-Geisser corrected; SE = 0.01; B =  + ∞). Bonferroni corrected comparisons revealed that bereavement was higher for the emotional-recognition metric for sadness compared to melancholy (*p* < 0.001; d = 1.86), misery (*p* < 0.001; d = 1.23) and despair (*p* < 0.001; d = 1.21). Misery (*p* < 0.001; d = 1.16) and despair (*p* < 0.001; d = 1.26) were higher than melancholy. Misery and despair did not provide evidence for being significantly different and provided Bayesian evidence for proximate emotional-recognition scores (*p* = 0.99; d = 0.11; SE = 0.01; B = 0.31). No gender or session sequence effects were reported (see supplementary material 5). These findings suggested bereavement was possibly the closest assessed emotional state to prototypical expressions of sadness in the current design. Overall, the current findings suggested there were discernible physiological and facial-emotional expressive differences between emotional faces showing melancholy, misery, bereavement and despair (see Table 1).

**Table 1:** Physiological and facial-emotional expressive responses to emotional faces

Mean (SD)					
	Melancholy		Misery	Bereavement	Despair
Skin conductance Responses (μS)	.19 (.05)	.35 (.06)		.34 (.06)	.51 (.09)
Heart-rate Responses (bpm)	2.05 (.62)	3.41 (.64)		3.42 (.65)	5.71 (1.08)
Facial-Emotional Recognition Metric (%)	.66 (.07)	.72 (.04)		.79 (.07)	.72 (.04)

Physiological responses including SCR and heart-rate responses, and facial-emotional expressive assessment for the selected faces expressing melancholy, misery, bereavement and despair

#### Results and discussion: IAPS images

Skin conductance responses for melancholy, misery, bereavement and despair were significantly different (F (2.49, 164.95) = 777.45; *p* < 0.001; η^2^_p_ = 0.92; Greenhouse-Geisser corrected; SE = 0.01; B =  + ∞). Bonferroni corrected comparisons revealed that despair was higher for SCR compared to melancholy (*p* < 0.001; d = 2.82), misery (*p* < 0.001; d = 1.91) and bereavement (*p* < 0.001; d = 1.82). Misery (*p* < 0.001; d = 2.5) and bereavement (*p* < 0.001; d = 2.75) were also higher than melancholy. Misery and bereavement did not provide evidence for being significantly different (*p* = 0.98; d = 0.03) and provided a trend for Bayesian evidence for proximate SCR changes (SE = 0.01; B = 0.58). Similar results were revealed for heart-rate responses (F (3,198) = 497.74; *p* < 0.001; η^2^_p_ = 0.88; SE = 0.02; B =  + ∞). Bonferroni corrected comparisons revealed that despair was higher for HR compared to melancholy (*p* < 0.001; d = 6.66), misery (*p* < 0.001; d = 3.73) and bereavement (*p* < 0.001; d = 3.49). Misery (*p* < 0.001; d = 2.72) and bereavement (*p* < 0.001; d = 2.97) were higher than melancholy. Misery and bereavement did not provide evidence for being significantly different for HR changes (*p* = 0.98; d = 0.02; SE = 0.11; B = 1.92). Participants exhibited expressions of sadness involving differences for emotional recognition (F (2.58, 170.27) = 25.15; *p* < 0.001; η^2^_p_ = 0.28; Greenhouse-Geisser corrected; SE = 0.01; B =  + ∞). Bonferroni corrected comparisons revealed that bereavement was higher for emotional recognition for sadness compared to melancholy (*p* < 0.001; d = 1.96), misery (*p* < 0.001; d = 0.92) and despair (*p* < 0.001; d = 0.71). Misery (*p* < 0.001; d = 0.71) and despair (*p* < 0.001; d = 0.79) were higher than melancholy. Misery and despair did not provide evidence for being significantly different and provided Bayesian evidence for proximate emotional-recognition scores (*p* = 0.99; d = 0.13; SE = 0.01; B = 0.33). No differences in specific action units were found between the four emotional states. No gender or session sequence effects were reported (see supplementary material 5). Bereavement was again the most effective elicitor of sad emotional expressions from the pool of the assessed emotional states. These findings suggested there were discernible differences between images showing melancholy, misery, bereavement and despair (see Table 2).

**Table 2:** Physiological facial-emotional expressive responses to emotional images

Mean (SD)					
	Melancholy		Misery	Bereavement	Despair
Skin conductance Responses (μS)	.17 (.04)	.27 (.04)		.26 (.05)	.48 (.02)
Heart-rate Responses (bpm)	1.59 (.36)	2.68 (.38)		2.78 (.39)	4.23 (.43)
Facial-Emotional Recognition Metric (%)	.61 (.04)	.65 (.07)		.71 (.05)	.66 (.08)

Physiological responses including SCR and heart-rate responses, and facial-emotional expression responses for the selected images expressing melancholy, misery, bereavement and despair

### Stage three: Matching task

#### Aims

The aim of this stage was to explore whether images from the International Affective Picture System can be matched with emotional faces which were selected as expressing the same emotional state. Our exploratory hypothesis for this stage was that participants would be able to associate IAPS elicitors with same-labelled faces.

#### Participants

A power calculation based on medium effect sizes (η^2^_p_ = 0.06; f = 0.25) and within-subject experimental trial repetitions was performed. The result revealed ninety-one participants would be required for P _(1-β)_ ≥ 0.9; (*p* ≤ 0.05; P (H_0_) ≥ 0.9; B < 0.33; η^2^_p_ [0, < 0.001]). The exclusion criteria for this stage were identical with phases one and two in stage one, and stage two. Ninety-four volunteers participated in this stage. Data from one participant were excluded from the analyses due to a self-report for having previously attended an experimental session including IAPS images (see Lang & Bradley, 2007; pp. 171–173). Data from two participants were excluded from the analyses due to the loss of a close family member in the last six months. The final sample consisted of ninety-one participants (forty-seven female) with average age 29.81 (SD = 4.11). No participant from stages one and two was included in the current stage. The experiment was approved by the Ethics Committee of the School of Psychology of the University of Nottingham.

#### Participant assessment

**Fig. 4:**
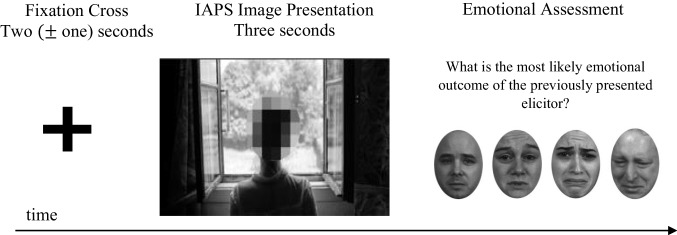
Experimental sequence for stage three. Example experiment sequence for stage three. Participants were presented with the option “other” without an accompanying image during the Emotional Assessment. They were asked to choose an item by clicking on it and press “OK” in the bottom of the screen to confirm their choice. The positioning of the stimuli during the Emotional Assessment was randomised in each trial. Each IAPS image and emotional face was presented once in each experimental session

The stimuli for this phase were presented using the same basic instructions, equipment and apparatus, and were coded in the same platform as stages one and two. Five random IAPS images per emotional state and twenty emotional faces per emotional state were presented during this stage. The experiment started with a training stage during which participants familiarised themselves with the mouse response components and the terminology of the experiment. The main experiment started with a fixation cross for two (± one) seconds. After the fixation cross a single random IAPS image showing melancholy or misery, or bereavement or despair was presented at fixation for three seconds. The image was not labelled. A blank screen interval was then presented for two seconds. After the interval participants were presented simultaneously in a lined arrangement with random faces showing melancholy, misery, bereavement, despair (non-labelled) and a non-facial option quoting “other”, and they were asked from an on-screen message “What is the most likely emotional outcome of the previously presented elicitor?”. The participants were asked to use the mouse to select an item and confirm their choice by pressing “OK” at the bottom of the screen. The positioning of the stimuli was randomised in each trial. Each IAPS image and emotional face was presented once in each experimental session (see Fig. 4).

#### Results and discussion

**Fig. 5:**
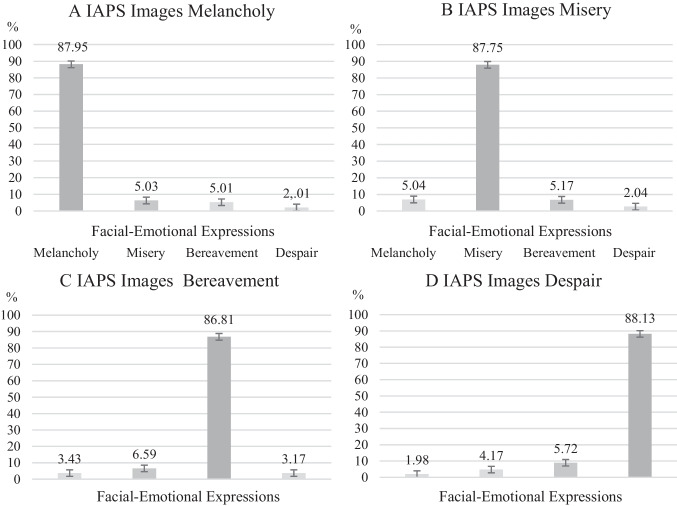
Responses (%) for matching IAPS emotional elicitors and emotional expressions. Responses in percentage for melancholy (**A**), misery (**B**), bereavement (**C**) and despair (**D**) for stage three for matching IAPS emotional elicitors to facial-emotional expressions that were selected as the same and different emotional states. Bars indicate ±2 standard errors of the mean

To explore whether IAPS images could be matched with facial-emotional outcomes, a repeated measures ANOVA was run with independent variables Emotional IAPS Elicitors (Melancholy, Misery, Bereavement & Despair) and Facial-Emotional Outcomes (Melancholy, Misery, Bereavement & Despair) and matching responses (%) as the dependent variable. The analyses revealed there were no significant differences and there were Bayesian evidence for proximate variation in responses between Emotional IAPS Elicitors (F (3, 270) = 1.23; *p* = 0.29; η^2^_p_ = 0.01; SE = 0.02; B = 0.29), the analyses also revealed a significant effect of Facial-Emotional Outcomes (F (3, 270) = 4.14; *p* ≤ 0.01; η^2^_p_ = 0.04; SE = 0.02; B =  + ∞) and a significant Emotional IAPS Elicitor to Facial Emotional Outcomes interaction (F (9, 810) = 1,528.91; *p* ≤ 0.001; η^2^_p_ = 0.94; SE = 0.02; B =  + ∞). Further analyses revealed that IAPS elicitors associated with melancholy (F (1.93, 174.01) = 1,389.85; *p* ≤ 0.001; η^2^_p_ = 0.94; Greenhouse-Geisser corrected; SE = 0.03; B =  + ∞) were significantly higher for choosing melancholic faces compared to misery (*p* < 0.001; d = 8.24), bereavement (*p* < 0.001; d = 7.19) and despair (*p* < 0.001; d = 9.26) after applying Bonferroni corrections for multiple comparisons. The same effect was reported for misery (F (2.07, 186.03) = 883.52; *p* ≤ 0.001; η^2^_p_ = 0.91; Greenhouse-Geisser corrected; SE = 0.04; B =  + ∞) with higher rates for matching misery-related IAPS images to misery-related emotional faces compared to melancholy (*p* < 0.001; d = 5.29), bereavement (*p* < 0.001; d = 7.16) and despair (*p* < 0.001; d = 8.56). Bereavement provided a similar pattern of results (F (1.91, 171.45) = 1,777.97; *p* ≤ 0.001; η^2^_p_ = 0.94; Greenhouse-Geisser corrected; SE = 0.02; B =  + ∞) compared to melancholy (*p* < 0.001; d = 7.09), misery (*p* < 0.001; d = 7.57) and despair (*p* < 0.001; d = 8.33). Finally, despair (F (1.68, 151.01) = 1098.01; *p* ≤ 0.001; η^2^_p_ = 0.92; Greenhouse-Geisser corrected; SE = 0.04; B =  + ∞) was also higher than melancholy (*p* < 0.001; d = 10.87), misery (*p* < 0.001; d = 10.06) and bereavement (*p* < 0.001; d = 5.21). These highly significant and very large in effect sizes outcomes were not surprising based on the thorough and rigorous methods for selecting IAPS images and facial-emotional elicitors during the previous stages, but also strongly suggested participants could match IAPS images and facial-emotional expressions that were selected as expressing the same emotional state, and that the assessed facial-emotional expressions involved specific emotional elicitors (see Fig. 5).

## Discussion

### Summary of findings

In the current set of studies, we explored whether sadness is associated and/or involves emotional states with discernible physiological correlates and eliciting characteristics. We provided – across a total of four experiments – evidence that supported these hypotheses and that supported that sadness is not a singular emotional condition. We showed that melancholy, misery, bereavement and despair were associated with sadness. We provided thorough empirical evidence that these emotional states involved dissociable experiential and physiological correlates and eliciting characteristics.

## General discussion

Previous research relating to the psychological assessment of sadness has provided diverse findings as regards its physiological and functional characteristics (Arias et al., 2020; Barrett et al., 2019). For example, a large body of research has proposed that sadness results in a decrease of peripheral nervous system arousal that enables self-reflection, planning and the revising of subsequent cognitive and behavioural attitudes and strategies (Power, 2010). Another equally substantial body of research has proposed that sadness involves an increase in peripheral nervous system arousal because it is associated with the distressing experience of failure (Rottenberg & Gross, 2003; Rottenberg et al., 2003) and the loss of an important object or subject (Saarimäki et al., 2018; Shirai & Suzuki, 2017).

Based on these, we tested the exploratory hypothesis that sadness is associated with potentially discernible emotional states. Our findings showed that previous findings in this area could be due to differences among emotional states associated with sadness (Arias et al., 2020; Barrett et al., 2019). We identified four distinguishable states associated with sadness. These were melancholy, misery, bereavement and despair. Each of these states involved specific physiological patterns, facial-expressive eliciting and scene-related eliciting characteristics.

For example, misery and bereavement provided Bayesian evidence for proximate valence and intensity ratings and, nevertheless, significant differences for physiological responses, emotional recognition and eliciting stimuli metrics, scene-related eliciting circumstances, and very high discriminability for matching same-labelled IAPS images to facial-emotional expressions. The same pattern applied to the ability of the participants to attribute expressions of melancholy and despair to corresponding IAPS images.

Conversely, the selected facial elicitors chosen as expressing melancholy involved several more pronounced prototypical sadness facial-expression characteristics, such as higher intensity for action units four (brow lowered) and 23 (lip tightener), compared to the selected facial elicitors for despair, although despair was rated significantly higher for intensity and negative valence (see Fig. 2C). Higher expressive characteristics associated with prototypical sadness were again revealed for selected facial elicitors for misery and bereavement, such as higher intensity for action units one (lowered brow raise) and 15 (lip corner depressor), compared to despair although despair was higher in intensity and negative valence than the selected elicitors for misery and bereavement (see Fig. 2B & C).

A challenging finding of the current research was that the selected faces for despair provided evidence for unexpected facial-emotional characteristics (Swann, 1992). Despair was the most arousing emotional state for both skin conductance responses and heart rate changes. As a selected facial elicitor, it included facial movements, such as pronounced head lowering stances (AU27/54) and closed eyes (AU44), that were the most uncharacteristic to prototypical sadness (see Fig. 2). Despair was most commonly elicited by IAPS images depicting irreparable catastrophe on a large scale (see Fig. 3). The latter could mean that despair could also be associated with emotions such as fear, and it could relate to helplessness and inundated personal defeat (Brown & Dutton, 1995).

It is noteworthy that faces expressing melancholy involved the most instances of wandering-averted gaze. Misery and bereavement included mainly direct eye contact while despair on most occasions involved closed eyes (90% of stimuli) or a gaze directed to the floor (10% of stimuli). This could be interpreted to suggest that melancholy is an inward-directed emotion that relates to mind-wandering and reminiscence, misery and bereavement could reflect trying to come to terms with witnessing or experiencing a painful and sorrowful occurrence while despair could relate to helplessness and witnessing irreparable catastrophe on a large scale (Hietanen, 2018; Dalmaso et al., 2020; see Fig. 3A).

These findings illustrate an opposite pattern of outcomes than these that we would expect if these states were linear escalations in the intensity and negative valence of prototypical sadness. The current data suggest that the explored emotional states involved different physiological responses, functions, eliciting circumstances, and eliciting characteristics. These findings make the case that sadness is not a singular emotional state. They show that these emotional states associate with sadness but involve distinguishable characteristics.

These findings have potential clinical and further experimental applications that could be useful to clinical and social psychological research in this area (see for example Hallensleben et al., 2019). The explorations of these potentials were not part of the objectives of the current research. These potentials are mentioned as rally points for further empirical research. The overarching objectives of the current research were to explore whether we could provide evidence for the distinct and comparable physiological correlates and eliciting characteristics of several states, such as melancholy, misery, bereavement and despair, that previous research proposed as rally points for the exploration of emotional states associated with sadness (see Barrett et al., 2019). We were able to provide thorough empirical evidence that these differences in psychological correlates and distinct eliciting circumstances do exist, and we were able to provide support for previous reviews that proposed that sadness is neither a simple nor a singular emotional condition (see Arias et al., 2020). The current findings can motivate further research on the emotional characteristics that comprise the potentially multi-faceted and diverse correlates of sadness.

### Limitations

The data for this study, were collected prior to the legal self-isolation regulations related to COVID-19. In the current studies participants did not select loneliness as an emotional state associated with sadness. The participants selected loneliness as an elicitor for sadness and not a distinguishable emotional state associated with sadness (see Fig. 3). It is worth considering – after almost two years of partial to complete self-isolation – whether this finding will replicate currently at the end of the isolation period related to the pandemic. We explored a subset of emotional states that could be associated with sadness because they were explicitly referenced in previous reviews as potential rally points for further exploring sadness. It is extremely important and critical to communicate to the readership that combinations of the current characteristics and additional experiences/characteristics, such as guilt, nostalgy, regret, pain-and-relief, and even the elated discharge of tears after the achievement of a great and difficult feat, such as an important athletic achievement, could be emotional states associated with sadness (Arias et al., 2020; Barrett et al., 2019).

## Conclusions

Based on previous perspectives for possible diversities on the emotional characteristics of sadness, we explored, in the current manuscript, whether sadness could be associated with emotional states with differences in physiological responses and eliciting circumstances. We were able to empirically associate four distinguishable emotional states with sadness. These emotional states were melancholy, misery, bereavement and despair. Our findings showed that these were distinguishable emotional states associated with sadness. These findings motivate and contribute to further empirically exploring the psychological plurality of sadness.
